# Recent developments in scaling down and using single use probes for measuring the live cell concentration by dielectric spectroscopy

**DOI:** 10.1186/1753-6561-9-S9-P46

**Published:** 2015-12-14

**Authors:** John Carvell, Matthew Lee, Aditya R Bhat

**Affiliations:** 1 Aber Instruments Ltd, Aberystwyth, UK SY23 3AH

## Background

Dielectric spectroscopy (DS) has been used for over two decades to measure the live cell concentration of animal cells in bioreactors online. The technology measures the build up of charge around live cell membranes under the influence of an induced electric field. Dead cells, debris, air bubbles and solid media particles do not have the capability to hold charge around themselves and are hence, invisible to the technology. The capacitance thus measured is proportional to the live bio-volume of the culture. DS has been used extensively to monitor and control live cell concentrations in bioreactors from process development through to large scale production. For DS to be employed as a PAT tool, it is essential that it successfully measures cell concentration over a wide range of culture volumes. Utilising the same cell measurement technique through the entire scale up protocol can also be a distinct advantage. This paper describes the latest developments in dielectric spectroscopy, especially the scaling down of the technology for measurement of lower volume cultures (up to 1 ml) in real time. The paper will also illustrate the application of dielectric spectroscopy to single use bioreactors.

## Materials and Methods

### Upside-down probe

**Figure 1:**
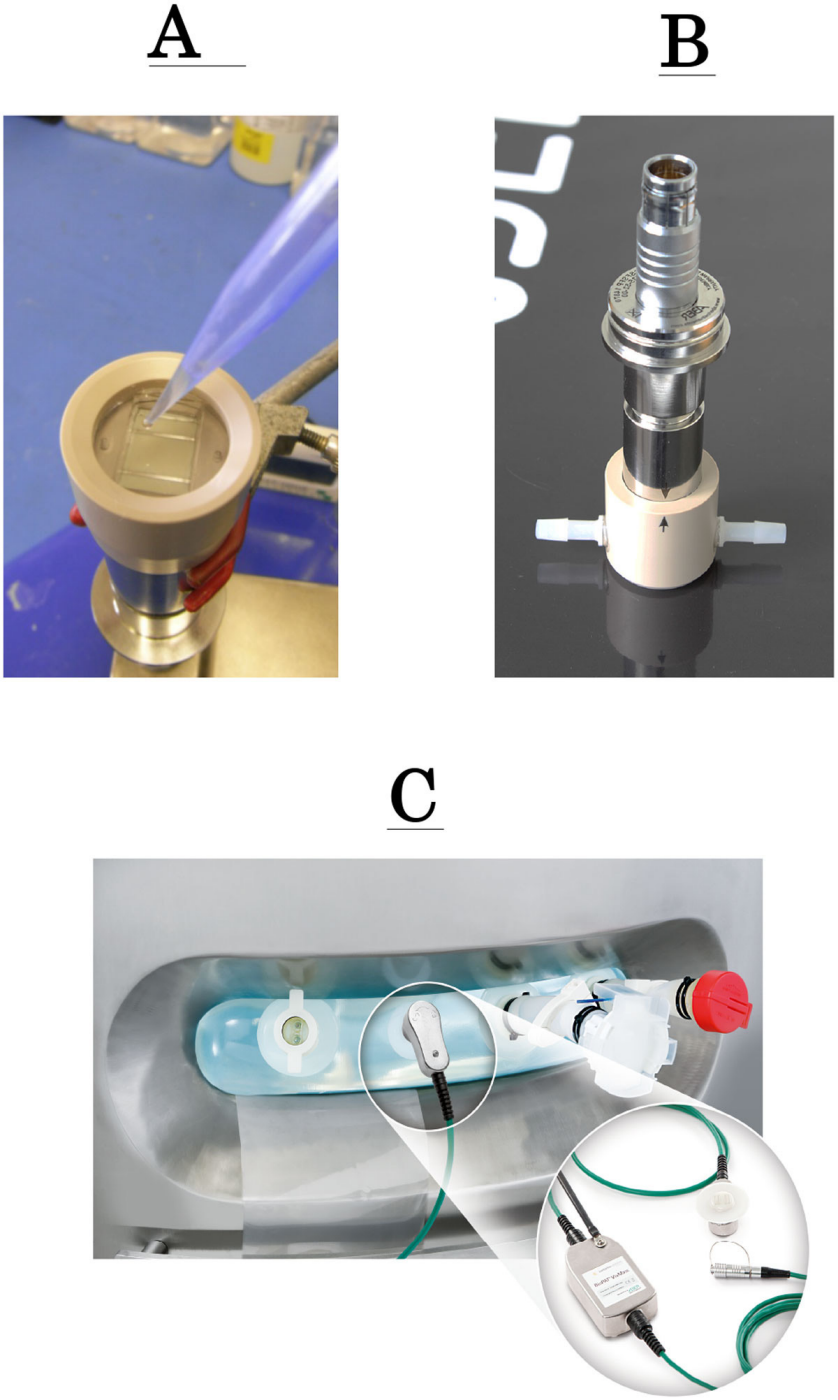
**Development in dielectric spectroscopy measurement by Aber Instruments Ltd A) Upside down probe B) Flow past cell C) Single-use probes**.

The "upside-down probe" (Figure 1A) was designed, in an effort to monitor live cell concentration in flask cultures. The probe is based on the robust 25 mm flush probe design normally used in production bioreactors. The probe is inverted upside down with a sample chamber attached on the flush surface. Samples down to 1 ml can be pipetted easily into the sample chamber for measurements in real-time. This makes it ideal to monitor cell concentration in shake flasks.

### Flow past cell

The "flow past cell" (Figure 1B) is based on the robust 25 mm flush probe design, much like the upside-down probe. A sleeve attached on top of the probe can be connected to appropriate silicone tubing. Therefore, it is possible to connect the flow past cell to a variety of bioreactors (SUB's or reusable) in the form of a recirculation loop. The entire assembly is designed to be autoclavable.

### 12 mm Annular probe

The 12 mm annular probe design by Aber Instruments can reliably measure cell concentration in smaller bioreactors down to a volume of 100 ml. The probe was installed in a DASGIP fully controllable bioreactor system and was used to measure a pluripotent stem cell batch culture (Hannover Medical School, Germany).

### Single Use Probe

The electrode geometry and platinum material that is exposed to cell culture in the single use probe is identical to the geometry and material used in the robust 25 mm diameter steam sterilizable probes with four flat, parallel flush electrodes. These single use probes have been proven to be ideal for the rocking motion bags and stirred tank bioreactors.

## Results

In an experiment to determine whether the 'upside down' probe is comparable to conventional Aber Instruments probes in bioreactors, it was seen that the data (not shown) from the two probes matched well.

It was crucial to determine the functioning of the flow past design within the normal working conductivity range of the Aber system. When the conductivity of water was increased from 1 mS/cm to 40 mS/cm, a very small change in capacitance was observed, thus proving the robustness of measurement using the flow past cell.

For experiments with the 12 mm annular probe, a heterogeneous rise/drop in pH pattern was observed during the run, depending on medium exchange. Although an overall increase in biomass was seen during the process, the Aber measurement helped observe a negative impact on live cell concentration as the pH dropped below 6.6; crucial information for process development.

When the live cell concentration measurement (courtesy Sartorius Stedim Biotech) using the disposable "disc" style probe (Figure 1C), with the Aber Mini Remote Futura, was compared with the measurement by the conventional reusable Aber probe for Chinese Hamster Ovary cells in Sartorius disposable bioreactors (RM 50L), it was seen that the capacitance measured by the two probes matched well with the wet cell weight and % viable biomass, even as the viability was seen to decrease towards the end of the culture.

The REACH consortium (a result of the FP7 StemExpand project) is developing an innovative bioreactor technology for the production of clinical-grade cell therapy products (hematopoietic and Mesenchymal Stem Cells). Measurement of cell concentration is pivotal for this project, since the aim is to achieve cell numbers in excess of 400 million cells/ml. In this effort, Aber Instruments is working with the REACH project, where a complete, integrated solution for measuring online viable biomass in the REACH single use bioreactor will be created.

## Conclusions

In conclusion, DS measurement (including frequency scanning) was successfully scaled down to measure sample volumes down to 1 ml, with important developments such as the upside-down probe and the flow past cell. DS can be used throughout the scale up process, right into production. Single-use probes, developed by Aber Instruments, have made it possible to monitor cell growth is SUB's online and in real time. Small single use biomass probes developed specifically for the REACH project will allow the DS technology to be applied to small scale bioreactors for stem cell production.
